# Bone health, cardiovascular disease, and imaging outcomes in UK Biobank: a causal analysis

**DOI:** 10.1093/jbmrpl/ziae058

**Published:** 2024-04-25

**Authors:** Dorina-Gabriela Condurache, Stefania D’Angelo, Ahmed M Salih, Liliana Szabo, Celeste McCracken, Adil Mahmood, Elizabeth M Curtis, Andre Altmann, Steffen E Petersen, Nicholas C Harvey, Zahra Raisi-Estabragh

**Affiliations:** NIHR Barts Biomedical Research Centre, William Harvey Research Institute, Centre for Advanced Cardiovascular Imaging, Queen Mary University of London, Charterhouse Square, London, EC1M 6BQ, England, United Kingdom; Barts Heart Centre, St Bartholomew’s Hospital, Barts Health National Health Service (NHS) Trust, West Smithfield, London EC1A 7BE, England, United Kingdom; MRC Lifecourse Epidemiology Centre, University of Southampton, Tremona Road, Southampton SO16 6YD, England,United Kingdom; NIHR Barts Biomedical Research Centre, William Harvey Research Institute, Centre for Advanced Cardiovascular Imaging, Queen Mary University of London, Charterhouse Square, London, EC1M 6BQ, England, United Kingdom; Department of Population Health Sciences, University of Leicester, Leicester LE1 7RH, England, United Kingdom; Department of Computer Science, Faculty of Science, University of Zakho, Zakho 42002, Kurdistan Region, Iraq; NIHR Barts Biomedical Research Centre, William Harvey Research Institute, Centre for Advanced Cardiovascular Imaging, Queen Mary University of London, Charterhouse Square, London, EC1M 6BQ, England, United Kingdom; Barts Heart Centre, St Bartholomew’s Hospital, Barts Health National Health Service (NHS) Trust, West Smithfield, London EC1A 7BE, England, United Kingdom; Semmelweis University, Heart and Vascular Centre, Budapest, Hungary; Division of Cardiovascular Medicine, Radcliffe Department of Medicine, National Institute for Health Research Oxford Biomedical Research Centre, University of Oxford, Oxford University Hospitals NHS Foundation Trust, Oxford OX3 9DU, England, United Kingdom; NIHR Barts Biomedical Research Centre, William Harvey Research Institute, Centre for Advanced Cardiovascular Imaging, Queen Mary University of London, Charterhouse Square, London, EC1M 6BQ, England, United Kingdom; Barts Heart Centre, St Bartholomew’s Hospital, Barts Health National Health Service (NHS) Trust, West Smithfield, London EC1A 7BE, England, United Kingdom; MRC Lifecourse Epidemiology Centre, University of Southampton, Tremona Road, Southampton SO16 6YD, England,United Kingdom; NIHR Southampton Biomedical Research Centre, University of Southampton and University Hospital Southampton NHS Foundation Trust, Southampton SO16 6YD, England, United Kingdom; Department of Medical Physics and Biomedical Engineering, Centre for Medical Image Computing (CMIC), University College London, London WC1E 6BT, England, United Kingdom; NIHR Barts Biomedical Research Centre, William Harvey Research Institute, Centre for Advanced Cardiovascular Imaging, Queen Mary University of London, Charterhouse Square, London, EC1M 6BQ, England, United Kingdom; Barts Heart Centre, St Bartholomew’s Hospital, Barts Health National Health Service (NHS) Trust, West Smithfield, London EC1A 7BE, England, United Kingdom; Health Data Research UK, British Heart Foundation Data Science Centre, London NW1 2BE, England, United Kingdom; MRC Lifecourse Epidemiology Centre, University of Southampton, Tremona Road, Southampton SO16 6YD, England,United Kingdom; NIHR Southampton Biomedical Research Centre, University of Southampton and University Hospital Southampton NHS Foundation Trust, Southampton SO16 6YD, England, United Kingdom; NIHR Barts Biomedical Research Centre, William Harvey Research Institute, Centre for Advanced Cardiovascular Imaging, Queen Mary University of London, Charterhouse Square, London, EC1M 6BQ, England, United Kingdom; Barts Heart Centre, St Bartholomew’s Hospital, Barts Health National Health Service (NHS) Trust, West Smithfield, London EC1A 7BE, England, United Kingdom

**Keywords:** heel ultrasound, bone health, cardiovascular diseases, cardiovascular magnetic resonance, Mendelian randomization, BMD, osteoporosis, cardiovascular imaging

## Abstract

This study examined the association of estimated heel bone mineral density (eBMD, derived from quantitative ultrasound) with: (1) prevalent and incident cardiovascular diseases (CVDs: ischemic heart disease (IHD), myocardial infarction (MI), heart failure (HF), non-ischemic cardiomyopathy (NICM), arrhythmia), (2) mortality (all-cause, CVD, IHD), and (3) cardiovascular magnetic resonance (CMR) measures of left ventricular and atrial structure and function and aortic distensibility, in the UK Biobank. Clinical outcomes were ascertained using health record linkage over 12.3 yr of prospective follow-up. Two-sample Mendelian randomization (MR) was conducted to assess causal associations between BMD and CMR metrics using genetic instrumental variables identified from published genome-wide association studies. The analysis included 485 257 participants (55% women, mean age 56.5 *±* 8.1 yr). Higher heel eBMD was associated with lower odds of all prevalent CVDs considered. The greatest magnitude of effect was seen in association with HF and NICM, where 1-SD increase in eBMD was associated with 15% lower odds of HF and 16% lower odds of NICM. Association between eBMD and incident IHD and MI was non-significant; the strongest relationship was with incident HF (SHR: 0.90 [95% CI, 0.89–0.92]). Higher eBMD was associated with a decreased risk in all-cause, CVD, and IHD mortality, in the fully adjusted model. Higher eBMD was associated with greater aortic distensibility; associations with other CMR metrics were null. Higher heel eBMD is linked to reduced risk of a range of prevalent and incident CVD and mortality outcomes. Although observational analyses suggest associations between higher eBMD and greater aortic compliance, MR analysis did not support a causal relationship between genetically predicted BMD and CMR phenotypes. These findings support the notion that bone-cardiovascular associations reflect shared risk factors/mechanisms rather than direct causal pathways.

## Graphical Abstract

**Graphical Abstract:**
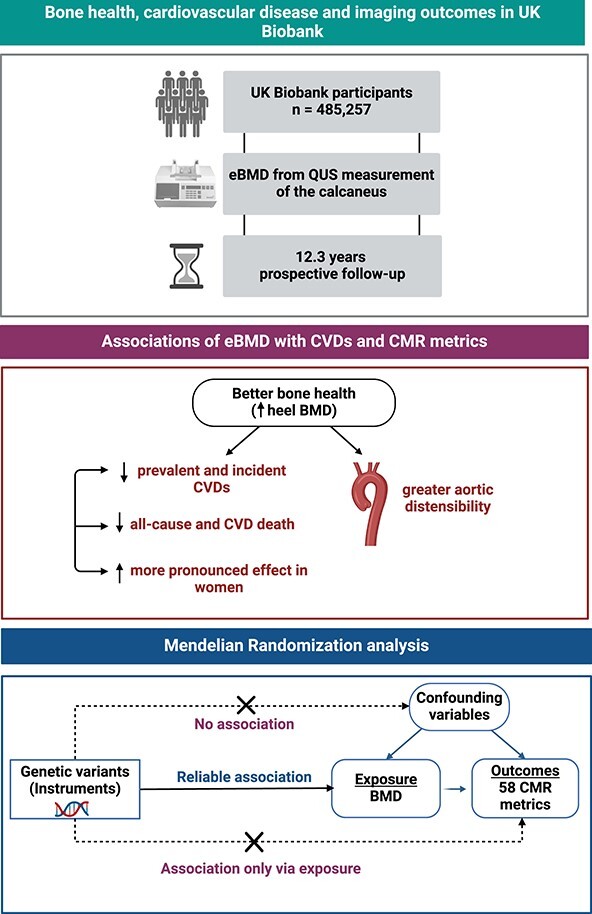

## Introduction

Cardiovascular diseases (CVDs) are the leading cause of mortality and a major contributor to disability worldwide.^1^ Osteoporotic bone fractures affect 1 in 2 women and 1 in 5 men over 50 yr, resulting in substantial long-term disability and reduced survival.^2^

Emerging epidemiological evidence suggests an association between osteoporosis and CVD outcomes.^3-8^ For instance, a recent prospective cohort study from the UK Biobank^3^ found that osteoporosis was strongly associated with cardiovascular mortality in men, with data suggesting a more than 2-fold increased risk of heart failure (HF) and coronary artery disease in those with osteoporosis.^4^

Osteoporosis and CVDs have a number of shared risk factors such as older age, sedentary lifestyle, tobacco use, excess alcohol intake, premature menopause, and vitamin D deficiency.^8^ Recently, an increasing body of biological and epidemiological evidence has provided support for a link between the 2 conditions beyond age and shared risk factors. It is suggested that a common pathogenic mechanism, including inflammation and imbalance in mineral metabolism, is implicated in their pathogenesis.^8^^,^^9^ Although associations between BMD and CVDs have been reported, there is unclear evidence regarding direct causal pathways between the two.

We present the most comprehensive evaluation of the relationship between bone and cardiovascular health in the UK Biobank. The aims of the present study were to explore the relationships of: (1) estimated heel bone mineral density (eBMD) with prevalent and incident CVDs and mortality events; (2) eBMD with cardiovascular magnetic resonance (CMR) measures of cardiac structure and function; (3) genetically predicted BMD with 58 CMR phenotypes using 2-sample Mendelian Randomization (MR) analysis.

To our knowledge, this is the first large-scale population-based study to examine the causal associations between BMD and cardiovascular health through detailed CMR phenotyping and MR analysis.

The utilization of CMR provides a highly sensitive and nuanced view of cardiovascular status capturing both clinically manifest diseases and pre-clinical cardiac alterations. This granularity enables a more precise assessment of the cardiac implications of BMD variations. By integrating the 2-sample MR analysis, our study seeks to provide a more definitive assessment of the causal effects of BMD on cardiovascular health, addressing a gap in the current understanding of these complex interrelations.

## Materials and methods

### Setting and study population

The UK Biobank includes over half a million individuals from across the United Kingdom (UK), aged 40–69 yr old at recruitment, which occurred over a 4-yr period between 2006 and 2010. Baseline assessment included a series of detailed questionnaires, face-to-face interviews, physical measures, and blood sampling.^10^ The UK Biobank Imaging Study, which includes CMR, is underway and aims to scan 100 000 of the original participants.^11^ Linkages to national health data, such as Hospital Episode Statistics (HES) and Office for National Statistics death registration data, permit prospective tracking of incident health events for all UK Biobank participants.

### Ascertainment of exposure

Heel eBMD was derived for all participants from QUS measurement of the calcaneus, using a Sahara Clinical Bone Sonometer (Hologic Corporation) according to a standardized protocol.^12^ The Sahara system measures the speed of sound (SOS, in m/s) and the broadband ultrasonic attenuation (BUA, in dB/MHz), which are used to estimate BMD (in g/cm^2^) per the manufacturer’s software. eBMD (in g/cm^2^) was derived as a linear combination of SOS and BUA (ie, eBMD = 0.002592 * (BUA + SOS) − 3.687). Vox software was used to automatically collect data from the sonometer (denoted direct input). In cases where direct input failed, quantitative ultrasound (QUS) outcomes were manually keyed into Vox by the attending healthcare technician or nurse (ie, manual input). QUS parameters are good predictors of fragility fractures and correlate reliably with BMD measured by DXA.^13-16^

### Ascertainment of clinical and mortality outcomes

The following prevalent and incident CVDs were included: ischemic heart disease (IHD), myocardial infarction (MI), HF, cardiomyopathies, atrial fibrillation (AF). Baseline date was the date each participant was recruited into the UK Biobank, from which their susceptibility to the events of interest was measured. Prevalent events were conditions present at baseline. Incident events were those occurring for the first time after baseline. Mortality outcomes (all-cause mortality, CVD mortality, IHD mortality) were defined according to the primary cause of death ascertained from death registration data. Individuals with record of the outcome of interest at baseline were excluded from the incident analyses for that condition. Diseases were defined based on a combination of UK Biobank baseline assessment records and HES International Classification of Disease codes (code set: Table SS1).

### Ascertainment of covariates

Covariates were selected based on their potential role as true confounders, determined from reported relationships with the exposure and outcome from published literature and biological plausibility. Age at baseline was used for models of prevalent and incident outcomes. Townsend deprivation index, a socio-economic measure of deprivation, was calculated prior to participants joining the UK Biobank based on area of residence. Educational level, alcohol intake frequency (daily or almost daily, 3–4 times per wk never, 1–2 times per wk, 1–3 times per mo, special occasions only, and never), smoking status (never smoker and current smoker), and physical activity (ascertained as duration of moderate physical activity [min/d)) were derived from self-report. BMI was calculated from height and weight measures taken at UK Biobank assessment. Diabetes, hypertension, and hypercholesterolemia status, at imaging, were defined based on self-report of the condition in UK Biobank assessments, self-reported of use of medication for the condition, or relevant ICD code in linked HES records (Table SS1).

### CMR image acquisition and analysis

CMR examinations were performed on 1.5 Tesla scanners (MAGNETOM Aera, Syngo Platform VD13A, Siemens Healthcare) in dedicated imaging units in accordance with predefined protocols.^17^ Images were analyzed using automated pipelines.^18^ The following CMR phenotypes were considered: left ventricular (LV) wall thickness (WT), LV mass (LVM), LV end-diastolic volume (LVEDV), LVM to LVEDV ratio, LV stroke volume (LVSV), LV ejection fraction, LV global functional index, LV global longitudinal strain, left atrial maximum volume, left atrial ejection fraction, right ventricle end-diastolic volume (RVEDV), right ventricle stroke volume, right ventricle ejection fraction (RVEF), aortic distensibility (AoD).

Alterations of CMR-derived metrics have known widely described significance in their relation to disease and prognosis. There is a large body of literature describing such relationships in clinical and population cohorts. Importantly, CMR may detect subclinical cardiovascular alterations before disease occurrence. For instance, greater LV mass has been highlighted as a poor prognostic marker across many studies,^18^^,^^19^ LV global longitudinal strain has been linked to poorer prognosis across a number of cohorts,^20^^,^^21^ and arterial stiffness (as indicated by lower aortic compliance) has a well-established linked to greater IHD risk.^22^^,^^23^

### Mendelian randomization

Two-sample MR was conducted to assess causal association between genetically predicted BMD and CMR metrics. We reviewed existing literature to identify genome-wide association studies (GWAS) capturing BMD (exposure) and CMR phenotypes (outcome). We ensured comparability of the exposure and outcome GWAS populations and that there was no overlap in cohorts between the two. Notably, the eBMD GWAS was not used due to the complete sample overlap, which can bias the MR estimates. From the identified GWAS studies, we selected suitable genetic instruments required for a 2-sample MR analysis.

#### Genome-wide association studies BMD (exposure)

Medina-Gomez et al.^24^ conduced a meta-analysis GWAS for total body (TB) BMD, including a total of 66 628 individuals from 30 cohorts across Europe, Australia, and America, comprising mostly individuals from European ancestry (86%).

#### GWAS cardiac function and structure (outcomes)

A total of 58 CMR measures of cardiac function and structure from 7 studies were considered. All studies used the UK Biobank to calculate the cardiac measures and conduct the GWAS. There were differences in sample size and quality control criteria across studies. Due to these variations, we have limited the analysis to metrics that were included in more than one study. For additional information regarding the studied included, refer to Table SS2 in the Supplemental material.

#### Selection of instrumental variables

The instrumental variables were selected from the BMD GWAS including the result of GWAS when all individuals were considered. We chose variants that passed the GWAS standard *P*-value threshold (*P* < 5 × 10^−8^). Then, we applied linkage disequilibrium (LD) clumping to choose independent variants. GWAS summary statistics were extracted from variants that passed both the GWAS *P*-value threshold and LD clumping (windows size = 10 000, r^2^ threshold = 0.001, population = European). We employed SNPs, with a comprehensive list and detailed information on each variant in Table SS3. The SNPs were derived from TB BMD and were all present in the outcome; however, 4 SNPs (rs11995824, rs2553773, rs447911, and rs780096) were excluded due to palindromicity, leaving 81 SNPs in the analysis. The scope of our association analysis did not extend to the establishment of minor allele frequency (MAF) ranges or imputation quality thresholds for SNP inclusion; however, comprehensive MAF and imputation quality data can be found in the study by Medina-Gomez et al.^24^

For the same set of variants, GWAS summary statistics for cardiac metrics were extracted. Thereafter**,** 2-sample MR was conducted to assess the causal association between BMD (Non-UK Biobank) as exposure and the 58 cardiac function and structure metrics (from UK Biobank) as outcome. Inverse variance weighted (IVW) method was used as the main analysis, while MR-Egger, weighted median, and weighted mode were used as complementary sensitivity analyses to detect direct and horizontal pleiotropy. Estimates of pleiotropy (Egger intercept) and heterogeneity are found in Table SS4. The analysis was conducted using the R package TwoSampleMR.^25^

### Statistical analysis

Statistical analysis was performed using RStudio V.4.1.0 (https://www.R-project.org/) and Stata V.17.^26^ Baseline characteristics are presented as number (percentage) for categorical variables, mean (SD) for normally distributed continuous variables, and median (IQR) for non-normally distributed continuous variables. Logistic regression and competing risk regression were used to estimate association of heel eBMD with prevalent and incident CVDs, respectively. The results are reported as odds ratios (ORs) and sub-distribution HRs (SHR) per 1-SD increment of eBMD and 95% CIs. The censor date was September 30, 2021, providing an average prospective follow-up of 12.3 yr. Current analysis does not account for multiple testing. The application will attenuate some of the already weak associations. This is in keeping with the notion that the relationship between BMD and CVD outcomes is small.

We estimated association of baseline heel eBMD with mortality outcomes using Cox regression models and the results are reported as hazard ratio (HRs) per 1-SD increment of BMD. In participants with CMR data available, we used multivariable linear regression to estimate the associations of heel eBMD with selected cardiovascular phenotypes. Associations of eBMD with CMR metrics are reported as SD change in CMR measure per 1-SD increment in eBMD. To allow comparison of the magnitude of effects across CMR metrics, we report standardized beta-coefficients with corresponding 95% CI.

We created 3 models with different layers of adjustment. Model 1 was adjusted for age and sex; model 2 was adjusted for model 1 variables plus diabetes, hypertension, high cholesterol, smoking status, alcohol consumption frequency, physical activity, Townsend deprivation score, education. Our fully adjusted model, model 3, was adjusted for model 2 variables plus BMI. To examine potential sex differential relationships, we report *P*-values for sex interaction terms (sex × eBMD) in fully adjusted models (model 3) and present sex stratified analyses for each outcome.

## Results

### Baseline characteristics

Baseline eBMD was available for 485 257 participants. At the time of recruitment, their mean age was 56.5 ± 8.1 yr and 54.5% of the participants were women. Mean eBMD was 0.55 (SD 0.14) g/cm^2^. Baseline participant characteristics are summarized in Table 1.

**Table 1:** Baseline characteristics of men and women with heel eBMD measured at baseline.

	**Whole set (*n* = 485 257)**	**Women (*n* = 264 762)**	**Men (*n* = 220 495)**
Age, mean (SD)	56.5 (8.1)	56.3 (8.0)	56.7 (8.2)
Townsend score, median (IQR)	−2.2 (−3.7,0.5)	−2.2 (−3.6,0.4)	−2.2 (−3.7,0.6)
Educational level, *N* (%)			
College/University	156 906 (32.3)	82 429 (31.1)	74 477 (33.8)
Other professional qualification/A levels	158 651 (32.7)	83 130 (31.4)	75 521 (34.3)
GCSE or less	163 963 (33.8)	96 196 (36.3)	67 767 (30.7)
Missing	5737 (1.2)	3007 (1.1)	2730 (1.2)
Physical activity (min/d), median (IQR)	30 (15,60)	30 (15,60)	30 (15,60)
Body mass index (kg/m^2^) mean (SD)	27.4 (4.8)	27.1 (5.2)	27.8 (4.2)
Alcohol intake frequencies, *N* (%)			
Daily or almost daily	98 473 (20.3)	42 588 (16.1)	55 885 (25.4)
Three or 4 times a week	111 919 (23.1)	54 379 (20.5)	57 540 (26.1)
Once or twice a week	125 237 (25.8)	68 162 (25.7)	57 075 (25.9)
One to 3 times a mo	53 995 (11.1)	34 424 (13.0)	19 571 (8.9)
Special occasions only	55 726 (11.5)	39 668 (15.0)	16 058 (7.3)
Never	38 871 (8.0)	25 008 (9.5)	13 863 (6.3)
Missing	1036 (0.2)	533 (0.2)	503 (0.2)
Current smoking status, *N* (%)			
No	433 592 (89.4)	240 822 (91.0)	192 770 (87.4)
Yes, most days	37 589 (7.8)	17 982 (6.8)	19 607 (8.9)
Only occasionally	13 200 (2.7)	5489 (2.1)	7711 (3.5)
Missing	879 (0.2)	469 (0.2)	407 (0.2)
Diabetes, *N* (%)	44 950 (9.3)	18 668 (7.1)	26 282 (11.9)
Hypertension, *N* (%)	187 453 (38.6)	89 913 (34.0)	97 540 (44.2)
High cholesterol, *N* (%)	119 417 (24.6)	50 562 (19.1)	68 855 (31.2)
Heel eBMD, mean (SD)	0.55 (0.14)	0.52 (0.12)	0.58 (0.15)
**Prevalent CVDs**			
Ischemic heart disease, *N* (%)	23 699 (4.9)	7781 (2.9)	15 918 (7.2)
Myocardial infarction, *N* (%)	11 201 (2.3)	2203 (0.8)	8998 (4.1)
Heart failure, *N* (%)	2180 (0.5)	538 (0.2)	1642 (0.7)
Cardiomyopathies, *N* (%)	821 (0.2)	223 (0.1)	598 (0.3)
Atrial fibrillation, *N* (%)	2474 (0.5)	807 (0.3)	1667 (0.8)
**Incident CVDs** ^**^a^**^			
Ischemic heart disease, *N* (%)	32 408 (6.7)	12 137 (4.6)	20 271 (9.2)
Myocardial infarction, *N* (%)	10 106 (2.1)	3182 (1.2)	6924 (3.1)
Heart failure, *N* (%)	13 957 (2.9)	5236 (2.0)	8721 (4.0)
Cardiomyopathies, *N* (%)	2791 (0.6)	1158 (0.4)	1633 (0.7)
Cardiac arrhythmia (Atrial fibrillation), *N* (%)	23 149 (4.8)	8911 (3.4)	14 238 (6.5)
**Mortality**			
All-cause, *N* (%) CVD, *N* (%) IHD, *N* (%)	35 950 (7.4) 13 073 (2.7) 4146 (0.9)	14 734 (5.6) 4243 (1.6) 863 (0.3)	21 216 (9.6) 8830 (4.0) 3283 (1.5)
**CMR metrics^b^ (min *N* = 25 320)**			
LV WT (mm), mean (SD)	5.7 (0.8)	5.2 (0.5)	6.2 (0.7)
LVM (g), mean (SD)	86.2 (22.2)	70.9 (12.2)	102.8 (18.3)
LVEDV (mL), median (IQR)	143.9 (123.5168.4)	127.4 (113.8143.2)	166.0 (147.0,187.6)
LVM: LVEDV (g/mL), median (IQR)	0.57 (0.52,0.63)	0.54 (0.50,0.59)	0.61 (0.56,0.67)
LVSV (mL), mean (SD)	87.7 (19.2)	79.0 (14.4)	97.2 (19.3)
LVEF (%), mean (SD)	59.6 (6.1)	62.2 (5.6)	57.8 (6.1)
LV GLS (%), mean (SD)	−18.5 (2.7)	−19.1 (2.7)	−17.8 (2.6)
LAV max (mL), median (IQR)	70.0 (57.0,85.3)	65.9 (54.4,78.7)	75.9 (61.0,92.6)
LAEF (%), mean (SD)	61.3 (9.1)	61.9 (8.6)	60.5 (9.7)
RVEDV (mL), mean (SD)	156.6 (37.3)	134.2 (24.3)	181.2 (33.3)
RVSV (mL), mean (SD)	89.1 (20.3)	79.4 (15.1)	99.8 (19.9)
RVEF (%), mean (SD)	57.3 (6.1)	59.3 (5.7)	55.2 (5.9)
AoD (10^−3^ mmHg^−^), median (IQR)	2.2 (1.5,3.0)	2.2 (1.5,3.1)	2.2 (1.6,3.0)

a People with a pre-existing record of each condition have been excluded.

b AoD, aortic distensibility; CMR, cardiovascular magnetic resonance; CVD, cardiovascular disease; eBMD, estimated bone mineral density; GCSE, general certificate of secondary education; IHD, ischemic heart disease; IPAQ, international physical activity questionnaire; LAEF, left atrial ejection fraction; LAV, max, left atrial maximum volume; LVEDV, left ventricular end-diastolic volume; LVEF, LV ejection fraction; LV GLS, LV global longitudinal strain; LVM, left ventricular mass; LVM: LVEDV, LV mass to LV end-diastolic volume ratio; LVSV, LV stroke volume; LV WT, left ventricular wall thickness; MET, metabolic equivalent; RVEDV, right ventricle end-diastolic volume; RVEF, right ventricle ejection fraction; RVSV, right ventricle stroke volume.

Counts variables are presented as number (percentage), continuous variables as mean (SD) or median (IQR) based on distribution.

Within the whole sample, the most common prevalent CVDs were IHD, MI, and AF with rates of 4.9% (*n* = 23 699), 2.3% (*n* = 11 201), and 0.5% (*n* = 2474), respectively (Table 1). The least common prevalent CVDs were HF (*n* = 2180, 0.5%) and non-ischemic cardiomyopathy (NICM) (*n* = 821, 0.2%). The most common incident diseases were IHD (*n* = 32 408, 6.7%) and arrhythmia (*n* = 23 149, 4.8%). There were 10 106 (2.1%) incident MIs and 13 957 (2.9%) incident cases each of HF. Over a follow-up period of 12.3 yr, we observed 35 950 (7.4%) deaths; of these, 13 073 were attributed to CVD and 4146 to IHD (Table 1). CMR data were available for 25 320 participants. CMR phenotypes are presented in Table 1.

### Association of eBMD with prevalent disease

Within the entire cohort, higher heel eBMD was associated with decreased odds of all prevalent CVDs considered (Table 2). The greatest magnitude of effect was with HF and NICM, where 1-SD increase in eBMD was associated with 15% lower odds of HF (OR: 0.85 [95% CI, 0.81–0.90]) and 16% lower odds of NICM (OR: 0.84 [95% CI, 0.78–0.81] in the fully adjusted models. Higher eBMD was also associated with reduced odds of arrhythmia, MI, and IHD, but with very small effect sizes (HR: 0.95 to 0.97).

**Table 2:** Associations between baseline heel eBMD^a^ and prevalent CVDs^a^.

**Outcome**	**Model 1** ***n* = 485 257**	**Model 2** ***N* = 399 297**	**Model 3** ***n* = 399 297**
	*OR (95% CI)*		
Ischemic heart disease	0.99 (0.98,1.00)	0.98 (0.96,0.99)	0.97 (0.96,0.99)^b^
Myocardial infarction	0.96 (0.94,0.98)	0.96 (0.94,0.99)	0.96 (0.94,0.98)^b^
Heart failure	0.85 (0.82,0.89)	0.87 (0.82,0.91)	0.85 (0.81,0.90)^b^
Non-ischemic cardiomyopathies	0.86 (0.80,0.92)	0.85 (0.79,0.92)	0.84 (0.78,0.91)^b^
Arrhythmia	0.97 (0.93,1.01)	0.96 (0.92,1.00)	0.95 (0.91,0.99)^b^

a CVD, cardiovascular disease; eBMD, estimated bone mineral density.

b Denotes a statistically significant result.

Results are reported as odds ratios (ORs) per 1-SD increment of eBMD. Model 1 is adjusted for age and sex. Model 2 is adjusted for age, sex, diabetes, hypertension, high cholesterol, smoking status, BMI, alcohol intake frequency, physical activity, Townsend score, and educational level. Model 3: Model 2 + BMI.

In sex-stratified analyses, higher eBMD appeared to show a greater protective effect in women than men across all prevalent CVDs considered (Table SS5). We observed significant sex interaction in association with prevalent MI and arrhythmia outcomes (Table SS5). The inverse associations of eBMD with prevalent MI had greater magnitude of effect in women (OR: 0.9; 95% CI, 0.87–0.96) that men (OR: 0.97; 95% CI, 0.95–0.97). In associations between eBMD and prevalent arrhythmia, the relationship attenuated to null in men, but remained statistically significant in women (OR:0.88; 95% CI, 0.81–0.96).

### Association of eBMD with incident CVD and mortality events

**Figure 1:**
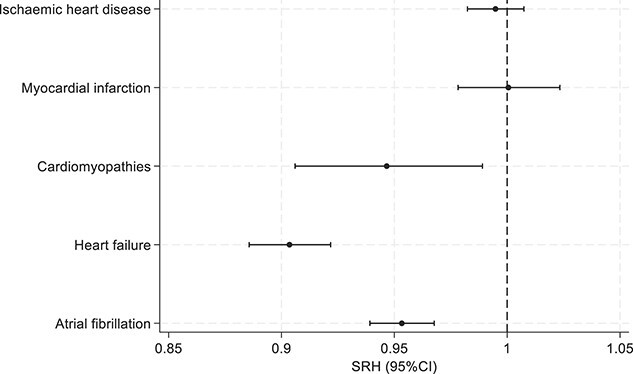
Associations between baseline heel eBMD and incident CVDs (*n* = 399 297). Footnote: Estimates are from model 3 adjusted for age, sex, diabetes, hypertension, high cholesterol, smoking status, BMI, alcohol intake frequency, physical activity, Townsend score, educational level, and BMI. The *x*-axis represents the sub-distribution hazard ratios (SHR) per 1-SD increment of eBMD obtained from Fine and Gray competing risk model. The *y*-axis lists incident cardiovascular diseases: IHD, MI, cardiomyopathies, HF, and AF.

**Figure 2:**
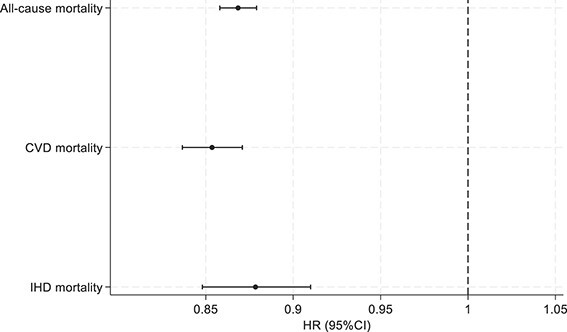
Association between baseline heel BMD and mortality events (*n* = 399 297). Footnote: Estimates are from model 3 adjusted for age, sex, diabetes, hypertension, high cholesterol, smoking status, BMI, alcohol intake frequency, physical activity, Townsend score, educational level and BMI. The *x*-axis represents the hazard ratios (HR) obtained with Cox proportional hazard model. The *y*-axis lists mortality events.

In fully adjusted models, higher eBMD was associated with lower risk of incident HF (SHR: 0.90 [95% CI, 0.89–0.92]), NICM (SHR: 0.95 [95% CI, 0.91–0.99]), and AF (SHR: 0.95 [95% CI, 0.94–0.97]) in the whole cohort (Table 3). Associations between eBMD and incident IHD and MI were statistically non-significant (Figure 1). Higher eBMD was consistently associated with a decreased risk of all-cause (HR: 0.87 [95% CI, 0.86–0.88]), CVD (HR: 0.85 [95% CI, 0.46–0.87]), and IHD (HR: 0.88 [95% CI, 0.45–0.91]) mortality, after adjusting for all relevant covariates (model 3). Detailed results of the multivariable Cox proportional hazard regression analyses are reported in Figure 2.

**Table 3:** Associations between baseline heel eBMD^a^ and incident CVDs^a^.

**Outcome**	**Model 1** ***n* = 485 257**	**Model 2** ***N* = 399 297**	**Model 3** ***n* = 399 297**
	*SHR (95% CI)*		
Ischemic heart disease	1.01 (0.99,1.02)	1.00 (0.98,1.01)	0.99 (0.98,1.01)
Myocardial infarction	1.00 (0.98,1.03)	1.00 (0.98,1.02)	1.00 (0.98,1.02)
Non-ischemic cardiomyopathies	0.96 (0.92,1.00)	0.95 (0.91,0.99)	0.95 (0.91,0.99)^b^
Heart failure	0.93 (0.91,0.95)	0.92 (0.90,0.94)	0.90 (0.89,0.92)^b^
Atrial fibrillation	0.98 (0.97,0.99)	0.97 (0.95,0.98)	0.95 (0.94,0.97)^b^

a CVD, cardiovascular disease; eBMD, estimated bone mineral density.

b Denotes a statistically significant result.

Results are reported as sub-distribution hazard ratios (SHR) per 1-SD increment of eBMD obtained from Fine and Gray competing risks model. Model 1 is adjusted for age and sex. Model 2 is adjusted for age, sex, diabetes, hypertension, high cholesterol, smoking status, alcohol intake frequency, physical activity, Townsend score, and educational level. Model 3: Model 2 + BMI.

**Figure 3:**
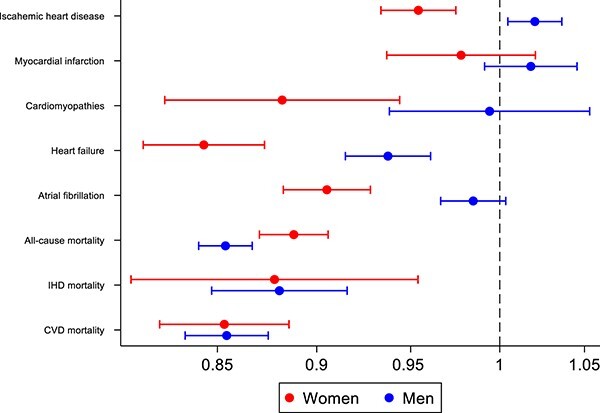
Sex-specific associations of heel eBMD with incident CVDs (SHR) and mortality events (HR): detailed estimates in Table SS6 and Table SS7 and model 3*.* Footnote: Estimates are from model 3 adjusted for age, sex, diabetes, hypertension, high cholesterol, smoking status, BMI, alcohol intake frequency, physical activity, Townsend score, educational level, and BMI. The *x*-axis represents the hazard ratios (HR) and sub-distribution hazard ratio (SHR). The *y*-axis lists incident CVDs and mortality events.

There was evidence of significant sex-specific associations between eBMD and all incident CVDs considered (Figure 3, Table SS6). Interaction terms with sex and eBMD, in fully adjusted models, were statistically significant for all incident CVDs included in our analysis. Higher eBMD appears to have a more protective relationship in women across all incident CVDs. Notably, in sex-stratified analyses, higher eBMD is associated with significantly lower risk of incident IHD (OR: 0.95; 95% CI, 0.93–0.98) and incident cardiomyopathies (OR: 0.88; 95% CI, 0.82–0.94) in women, while in men these relationships appeared statistically non-significant.

In the relationships with mortality events, the sex interaction term was statistically significant in relation to all-cause mortality (Figure 3, Table SS7). In sex-stratified analyses, higher eBMD was associated with significantly lower risk of all mortality outcomes (all-cause, CVD, IHD) in both men and women.

### Association of heel eBMD with CMR metrics

Higher heel eBMD was associated with greater AoD in fully adjusted linear regression models (β: 0.02 [95% CI, 0.009–0.04]). Associations with other CMR metrics were non-significant in fully adjusted models (Table 4).

**Table 4:** Association between baseline heel eBMD and CMR outcomes (exposure and outcomes are in SD).

	*β (95%CI)*		
**CMR metrics^a^**	**Model 1** ***n* = 485 257**	**Model 2** ***N* = 399 297**	**Model 3** ***n* = 399 297**
LVM (g)	0.03 (0.02,0.04)	0.03 (0.02,0.03)	0.009 (−0.0003,0.02)
LVEDV (mL)	0.02 (0.001,0.03)	0.01 (0.002,0.02)	0.002 (−0.009,0.01)
LVM: LVEDV (g/mL)	0.02 (0.01,0.03)	0.02 (0.01,0.04)	0.01 (−0.001,0.02)
LVSV (mL)	0.02 (0.01,0.03)	0.02 (0.004,0.03)	0.005 (−0.007,0.02)
LVEF (%)	0.001 (−0.01,0.01)	0.003 (−0.01,0.02)	0.003 (−0.01,0.02)
LV GLS (%)	−0.0005 (−0.01,0.01)	0.003 (−0.01,0.02)	0.01 (−0.005,0.02)
LAV max (mL)	0.01 (0.002,0.03)	0.007 (−0.01,0.02)	−0.01 (−0.02,0.01)
LAEF (%)	−0.01 (−0.03,0.001)	−0.006 (−0.02,0.01)	−0.004 (−0.02,0.01)
RVEDV (mL), mean (SD)	0.03 (0.02,0.04)	0.02 (0.01,0.04)	0.01 (0.003,0.02)
RVSV (mL), mean (SD)	0.02 (0.01,0.03)	0.02 (0.008,0.03)	0.009 (−0.003,0.02)
RVEF (%), mean (SD)	−0.02 (−0.03,−0.005)	−0.02 (−0.03,−0.002)	−0.01 (−0.03,−0.0003)
AoD (10^−3^ mmHg^−1^)	0.02 (0.003,0.03)	0.02 (0.01,0.03)	0.02 (0.009,0.04)^b^

a AoD, aortic distensibility; CMR, cardiovascular magnetic resonance; eBMD, estimated bone mineral density; LAV max, left atrial maximum volume; LAEF, left atrial ejection fraction; LVEDV, left ventricular end-diastolic volume; LVEF, LV ejection fraction; LVM, left ventricular mass; LVM: LVEDV, LV mass to LV end-diastolic volume ratio; LV GLS, LV global longitudinal strain; LVSV, LV stroke volume; RVEDV, right ventricle end-diastolic volume; RVEF, right ventricle ejection fraction; RVSV, right ventricle stroke volume.

b Denotes a statistically significant result.

Results are standardized beta coefficients (β) obtained with linear regression model. Model 1 is adjusted for age and sex. Model 2 is adjusted for age, sex, diabetes, hypertension, high cholesterol, smoking status, alcohol intake frequency, physical activity, Townsend score, and educational level. Model 3: Model 2 + BMI.

### MR analysis

The set of instrumental variables included 81 variants representing genetically predicted BMD, after applying GWAS *P*-value threshold and LD clumping. The results of the main analysis (Table SS4) indicate that 14 metrics (mostly from RV [eg, smaller RVEDV and RVESV] and 2 metrics from LV [Max LV and LVSV]) are significantly (IVW *P*-value <.05) influenced by BMD. However, the results of the sensitivity analysis did not confirm support for these association (*P*-value > .05), indicating a violation of the method’s assumptions (eg, direct or horizontal pleiotropy). Accordingly, this analysis did not provide evidence to support a potential causal relationship between BMD and CMR-derived cardiac function and structure measures.

## Discussion

### Summary of findings

We present the largest and most comprehensive evaluation of the relationship between bone and CV health in the UK Biobank. In this population-based cohort of 485 257 individuals, we examined the relationship between eBMD with prevalent and incident health outcomes, mortality (both all-cause and attributable to CVD and IHD), and CMR phenotypes. Although there were modest inverse associations between eBMD and CVDs events, the most significant associations were observed with mortality outcomes. Higher eBMD was associated with greater AoD; that is, better bone health was associated with better vascular health. However, associations with other CMR metrics were null. Furthermore, MR analysis did not support a causal link between genetically predicted BMD and a wide range of CMR phenotypes. Although there is an evident relationship between bone and CV health, our results indicate that this is most likely due to shared risk factors and common underlying biological processes rather than a direct causal effect. These findings provide insight into mechanistic pathways and inform long-term care and risk stratification considerations.

### Interpretation in the context of existing evidence

Our study found that individuals with higher heel eBMD were associated with a reduced risk of both prevalent and incident CVDs. This observation finds resonance with an established body of literature, which posits a relationship between BMD and CVD risk.^3-8^^,^^27^ For instance, a European epidemiological study suggests a 23% reduction in the risk of incident HF for every 1-SD increase in BMD.^28^ A systematic review and meta-analysis of 11 studies reported that individuals with low BMD had a 33% higher CVD risk.^29^ Although the available literature is consistent with our findings of links between BMD and CVDs, the notably larger effect sizes in these reports compared to our analysis likely reflect greater residual confounding, compared to our models, which included extensive confounder adjustment.

Sex-stratified sub-analysis revealed significant sex-specific associations between baseline heel eBMD and the incidence of various CVD outcomes. Higher eBMD was more protective against incident CVDs in women compared to men, particularly for IHD, HF, and arrhythmia. This is in contrast to the HUNT study,^30^ which showed a small protective association of BMD on MI and AF in men but not in the female population. Conversely, the research conducted by Yang et al.^4^ showed no gender difference between BMD, and the risk of CVD was observed in the sex-specific stratified analysis. Moreover, Gao et al.^31^ found that lower BMD was linked with higher risk of HF in older Black women and White men. The appreciation of sex differential relationships can be challenging, as this requires a much greater level of statistical powered. Sex-stratified analyses with CVD outcomes are prone to be differentially powered across men and women with propensity toward being underpowered in women who tend to have fewer events. Our analysis, including a large number of men and women and with over 12 yr prospective follow-up, had opportunity to capture adequate CVD events across both sexes, enhancing our ability to reliably detect sex-specific associations of eBMD. Furthermore, although reports in the literature are mixed, the greater influence of eBMD on cardiovascular health in women observed in our study is biologically consistent, particularly given the influence of menopause on both bone and cardiovascular health.

Associations of heel eBMD with mortality outcomes exhibit a larger effect size and demonstrate consistently statistically significant results across the different mortality outcomes considered, despite the previously described smaller associations with prevalent and incident IHD, respectively. This observation aligns with a growing body of evidence from observational studies over the past decade that has highlighted potential links between heel BMD and mortality events.^32-34^

The results of the main analysis confirm the association of better bone quality (higher eBMD) with better arterial health as reflected by higher AoD. In line with a recent study conducted in the same population,^35^ our findings not only replicate the observed inverse relationship between bone quality and arterial compliance as measured by CMR but also extend beyond, by examining a wider range of clinical outcomes, CMR parameters, and causal relationships using MR analysis.

To our knowledge this is the first large-scale population-based study to examine the causal associations between genetically predicted BMD and CMR phenotypes using 2-sample MR analysis. The findings do not support a causal link between genetically predicted BMD and CMR metrics, indicating that previously described observational relationships may be influenced by residual confounding rather than direct causality. Our study not only provides pivotal insights into the complex interplay between bone and cardiovascular health but also paves the way for future research to delve into the causality between genetically predicted BMD and cardiovascular outcomes. This will further enhance our understanding and inform both screening strategies and therapeutic interventions.

### Limitations

As the age range in UK Biobank was limited to 40 to 69 yr at recruitment, our results may not be applicable to individuals outside this age window. There is significant healthy and wealthy volunteer selection in the UK Biobank, which may limit generalizability of our findings.^36^^,^^37^ The exposure of interest (eBMD) was derived from QUS of the heel; however, DXA is the reference standard for assessment of BMD and diagnosis of osteoporosis in current guidelines.^38^^,^^39^ The UK Biobank imaging substudy includes DXA as part of its imaging protocol; however, the number of participants and duration of follow-up is currently limited for this subset. Thus, for the present analysis, eBMD was selected as the exposure of interest as it provided substantially greater statistical power of the order of many magnitudes. In future, studies with DXA may be considered as more data become available and more outcomes accrue.

## Conclusion

Higher BMD is linked to reduced risk of prevalent and incident CVD and mortality across a range of outcomes. Observational analyses further suggest associations between higher eBMD and better vascular health, as reflected by greater aortic compliance. MR does not support a causal relationship between BMD and cardiovascular structure and function across an extensive range of metrics. These findings support the notion that bone-cardiovascular associations reflect shared risk factors/mechanisms rather than direct causal pathways.

## Supplementary Material

Supplemental_material_clean_version_ziae058

## Data Availability

This project was carried out under UK Biobank Access Application 3593. UK Biobank will make the data available to all bona fide researchers for all types of health-related research that is in the public interest, without preferential or exclusive access for any persons. All researchers will be subject to the same application process and approval criteria as specified by UK Biobank. For more details on the access procedure, see the UK Biobank website: http://www.ukbiobank.ac.uk/register-apply.
