# Supplementary material for: Bone health, cardiovascular disease, and imaging outcomes in UK Biobank: a causal analysis
Source: JBMR Plus. 2024 Apr 25;8(6):ziae058. doi: 10.1093/jbmrpl/ziae058 (PMC11114472; doi:10.1093/jbmrpl/ziae058)
Supplement: Supplemental_material_clean_version_ziae058 [file supplemental_material_clean_version_ziae058.docx]

**Supplementary Material**

**Supplemental Table 1: ICD codes and UK Biobank field used to generate medical conditions**

| **Source** | **ICD code/UKB filed** | **Description** |
| --- | --- | --- |
| **Ischaemic heart disease (IHD)** | | |
| ICD9 | 4139 | Angina pectoris |
|  | 4140 | Coronary atherosclerosis |
|  | 4141 | Aneurysm of heart |
|  | 4148 | Other specified forms of chronic ischaemic heart disease |
|  | 4149 | Chronic ischaemic heart disease, unspecified |
|  | 4119 | Other acute and subacute forms of ischaemic heart disease |
| Self-report | 20002 | Angina: 1074 |
| ICD10 | I20 | Angina pectoris |
|  | I24 | Other acute ischaemic heart diseases |
|  | I25 | Chronic ischaemic heart disease |
| Diagnosed by doctor | 3627 | Age angina diagnosed |
|  | 6150: 2 | Angina |
| **Myocardial infarction (MI)** | | |
| ICD9 | 4109 | Acute myocardial infarction |
|  | 4129 | Old myocardial infarction |
| Self-report | 20002 | Heart attack/myocardial infarction |
| ICD9 | 410 | Acute myocardial infarction |
|  | 411 | Other acute and subacute forms of ischaemic heart disease |
|  | 412 | Old myocardial infarction |
| ICD10 | I21 | Acute myocardial infarction |
|  | I22 | Subsequent myocardial infarction |
|  | I23 | Certain current complications following acute myocardial infarction |
| Diagnosed by doctor | 3894 | Age heart attack diagnosed |
|  | 6150: 1 | Heart attack |
| **Non-ischaemic cardiomyopathies** | | |
| ICD9 | 4254 | Other primary cardiomyopathies |
| Self-report | 20002: 1079 | Cardiomyopathy |
|  | 20002: 1588 | Hypertrophic cardiomyopathy (HCM / HOCM) |
| ICD10 | I42 | Cardiomyopathy |
|  | I43 | Cardiomyopathy in diseases classified elsewhere |
|  | I11 | Hypertensive heart disease |
|  | I13 | Hypertensive heart and renal disease |
| **Heart failure (unspecified aetiology)** | | |
| ICD9 | 4280 | Congestive heart failure |
|  | 4281 | Left heart failure |
| Self-report | 20002: 1076 | Heart failure/pulmonary oedema |
| ICD10 | I50.0 | Congestive heart failure |
|  | I50.1 | Left ventricular failure |
|  | I50.9 | Heart failure, unspecified |
| **Cardiac arrhythmia (Atrial fibrillation)** | | |
| Self-report | 20002: 1471 | Atrial fibrillation |
| ICD9 | 4273 | Atrial fibrillation and flutter |
| ICD10 | I48.0 | Paroxysmal atrial fibrillation |
|  | I48.1 | Persistent atrial fibrillation |
|  | I48.2 | Chronic atrial fibrillation |
|  | I48.9 | Atrial fibrillation and atrial flutter, unspecified |
| **Diabetes** |  |  |
| Self-report | 20002 | Diabetes |
|  | 20002 | Type 1 diabetes |
|  | 20002 | Type 2 diabetes |
| Medications | 6177, 6153: 3 | Insulin |
| ICD9 | 250 | Diabetes mellitus |
| ICD10 | E10 | Type 1 diabetes mellitus |
|  | E11 | Type 2 diabetes mellitus |
|  | E13 | Other specified diabetes mellitus |
|  | E14 | Unspecified diabetes mellitus |
|  | G590 | Diabetic mononeuropathy |
|  | G632 | Diabetic polyneuropathy |
|  | H280 | Diabetic cataract |
|  | H360 | Diabetic retinopathy |
|  | M142 | Diabetic arthropathy |
|  | N083 | Glomerular disorders in diabetes mellitus |
|  | O240 | Diabetes mellitus in pregnancy: Pre-existing type 1 diabetes mellitus |
|  | O241 | Diabetes mellitus in pregnancy: Pre-existing type 2 diabetes mellitus |
|  | O243 | Diabetes mellitus in pregnancy: Pre-existing diabetes mellitus, unspecified |
|  | O244 | Diabetes mellitus arising in pregnancy |
|  | O249 | Diabetes mellitus in pregnancy, unspecified |
|  | Y423 | Insulin and oral hypoglycaemic [antidiabetic] drugs |
| Diagnosed by doctor | 2443 | Diabetes diagnosed by doctor |
|  | 2976 | Age diabetes diagnosed by doctor |
| **High cholesterol** |  |  |
| Self-report | 20002 | High cholesterol |
| Medications | 6177, 6153: 1 | Cholesterol lowering medication |
| ICD9 | 272 | Disorders of lipoid metabolism |
| ICD10 | E780 | Pure hypercholesterolaemia |
|  | E782 | Mixed hyperlipidaemia |
|  | E783 | Hyperchylomicronaemia |
|  | E784 | Other hyperlipidaemia |
|  | E785 | Hyperlipidaemia, unspecified |
| **Hypertension** |  |  |
| Self-report | 20002 | Essential hypertension |
|  | 20002 | Hypertension |
| Medications | 6177, 6153: 2 | Blood pressure medication |
| Diagnosed by doctor | 2966 | Age high blood pressure diagnosed |
|  | 6150: 4 | High blood pressure |
| ICD10 | I10 | Essential (primary) hypertension |
|  | I11.0 | Hypertensive heart disease with (congestive) heart failure |
|  | I11.9 | Hypertensive heart disease without (congestive) heart failure |
|  | I12.0 | Hypertensive renal disease with renal failure |
|  | I12.9 | Hypertensive renal disease without renal failure |
|  | I13.0 | Hypertensive heart and renal disease with (congestive) heart failure |
|  | I13.1 | Hypertensive heart and renal disease with renal failure |
|  | I13.2 | Hypertensive heart and renal disease with both (congestive) heart failure and renal failure |
|  | I13.9 | Hypertensive heart and renal disease, unspecified |
|  | I15.0 | Renovascular hypertension |
|  | I15.1 | Hypertension secondary to other renal disorders |
|  | I15.2 | Hypertension secondary to endocrine disorders |
|  | I15.8 | Other secondary hypertension |
| ICD9 | 4010 | Essential hypertension, specified as malignant |
|  | 4011 | Essential hypertension, specified as benign |
|  | 4019 | Essential hypertension, not specified as malignant or benign |
|  | 4039 | Hypertensive renal disease, not specified as malignant or benign |
|  |  |  |
|  |  |  |

**Supplemental Table1 footnote:** ICD10 codes are drawn from fields 41270, 41280, 41234 and 41259; ICD9 codes are drawn from fields 41271, 41281, 41234 and 41259; OPCS4 codes are drawn from fields 41272, 41282, 41149 and 41259.

**Supplemental Table 2: Published genome wide association studies from which the cardiovascular magnetic resonance variants were selected**

| **Paper** | **Sample size** | **Cardiac metrics** |
| --- | --- | --- |
| Francis CM et al.,^1^ | 32,590 | Descending aortic distensibility, maximum descending aortic area, ascending aortic distensibility, minimum ascending aortic area, minimum descending aortic area, maximum ascending aortic area |
| Aung N et al.,^2^ | 29,506 | RVESV, RVSV, RVEF, RVEDV |
| Aung N et al.,^3^ | 16 923 | LVEDV, LV mass, LV ESV (not included in our study), LVSV, LVEF, LV mass to EDV ratio |
| Fung K et al.,^4^ | 127,121 | Arterial stiffness index |
| Benjamins JW et al.,^5^ | 37,910 | Ascending aorta maximum area, ascending aorta minimum area, ascending aorta distensibility |
| Ahlberg G et al.,^6^ | 35 658 | LA max indexed, LA mini indexed, LA active emptying fraction, LA passive emptying fraction, LA total emptying fraction |
| Pirruccello JP et al.,^7^ | 40,000 | Max atrial area diameter Indexed, Short axis cm root Indexed, LVSV, LV Max, RVEDV, RVESV, Short axis cm PA Indexed, RVSV, Min LV, Short axis cm PA I, RA Max Indexed, Short axis cm PA, RVESV Indexed, Short axis cm PA diastole, RA Max, RA Min Indexed, Short axis cm root, Min LV I, Max atrial area diameter, RVEDV Indexed, RA Min, LVEF, LV Max, RVEF, RVSV/LVSV ratio, RVSV Indexed, RVEDV/LVEDV ratio, Short axis cm PA strain, PA/AO, LVSV Indexed, RVEF/LVEF ratio, PA/AO diastole, RVESV/LVESV ratio, Fractional area change |

**Supplemental Table 2 footnote:** Studies included in the Mendelian randomization analysis as outcomes. Indexed: to body surface area. cm, centimetre; PA, pulmonary artery; AO, aorta; RV, right ventricle; RVESV, RV end-systolic volume; RVSV, RV stroke volume; RVEF, right ventricular ejection fraction; RVEDV, right ventricular end-diastolic volume; LV, left ventricle; LVEDV, LV end-diastolic volume; LVESV, LV end-systolic volume; LVSV, LV stroke volume; LVEF, left ventricular ejection fraction; LA, left atria; LA max, maximum LA volume; LA min, minimum LA volume; RA, right atria; RA Max, maximum RA volume; RA min, minimum RA volume; LV Max, maximum LV volume.

**Supplemental Table 3: Single-Nucleotide Polymorphisms (SNPs) Included in the study**

| **Chr** | **Pos** | **rsid** | **EA** | **OA** | **EAF** | **beta** | **pval** | **N** | **StdErr** |
| --- | --- | --- | --- | --- | --- | --- | --- | --- | --- |
| 1 | 8422676 | rs2252865 | t | c | 0.3241 | -0.0328 | 4.72E-08 | 66075 | 0.006 |
| 1 | 22486029 | rs56104760 | a | g | 0.8095 | 0.0747 | 7.38E-24 | 66381 | 0.0074 |
| 1 | 22703035 | rs10493013 | t | c | 0.8189 | -0.1013 | 4.08E-43 | 66572 | 0.0074 |
| 1 | 68656697 | rs2566752 | t | c | 0.6103 | -0.0721 | 1.88E-34 | 66398 | 0.0059 |
| 1 | 68664913 | rs2566751 | a | t | 0.8727 | -0.0567 | 1.32E-08 | 66398 | 0.01 |
| 1 | 110475971 | rs7548588 | t | c | 0.609 | -0.0367 | 2.21E-10 | 66240.1 | 0.0058 |
| 1 | 172186729 | rs633995 | a | g | 0.4251 | 0.0351 | 1.61E-09 | 66119 | 0.0058 |
| 1 | 219897941 | rs4846580 | a | g | 0.5329 | 0.0345 | 3.21E-09 | 66614 | 0.0058 |
| 1 | 240581653 | rs12044944 | t | c | 0.1916 | 0.0553 | 7.54E-14 | 65125 | 0.0074 |
| 2 | 27741072 | rs780096 | c | g | 0.4406 | -0.0311 | 4.58E-08 | 66578 | 0.0057 |
| 2 | 40630678 | rs10490046 | a | c | 0.7609 | 0.0429 | 1.43E-10 | 65961 | 0.0067 |
| 2 | 42284110 | rs2289410 | a | t | 0.868 | 0.0494 | 2.00E-08 | 66494 | 0.0088 |
| 2 | 54684557 | rs11898505 | a | g | 0.3326 | 0.0342 | 1.28E-08 | 66091 | 0.006 |
| 2 | 68962137 | rs10048745 | a | g | 0.2477 | -0.0389 | 6.44E-09 | 66565.1 | 0.0067 |
| 2 | 85484818 | rs11904127 | a | g | 0.5512 | -0.0324 | 1.18E-08 | 66561 | 0.0057 |
| 2 | 119548256 | rs144279715 | a | g | 0.9853 | -0.2295 | 6.18E-15 | 64027 | 0.0294 |
| 2 | 119632252 | rs12612325 | a | g | 0.2122 | -0.0548 | 1.98E-12 | 66509 | 0.0078 |
| 2 | 166577489 | rs7586085 | a | g | 0.5337 | 0.0532 | 8.64E-21 | 66609 | 0.0057 |
| 2 | 202799604 | rs2350085 | t | c | 0.8726 | -0.0643 | 3.80E-14 | 66412 | 0.0085 |
| 2 | 202832130 | rs10931982 | t | c | 0.2097 | -0.0508 | 1.59E-08 | 55344.1 | 0.009 |
| 2 | 234303405 | rs838721 | a | g | 0.437 | -0.0314 | 4.48E-08 | 65515.9 | 0.0057 |
| 3 | 41127046 | rs447911 | c | g | 0.5348 | 0.0708 | 6.29E-36 | 66564 | 0.0057 |
| 3 | 156692207 | rs74394007 | a | c | 0.862 | 0.0608 | 2.46E-13 | 66607 | 0.0083 |
| 4 | 1006987 | rs76051363 | t | c | 0.1491 | -0.0794 | 1.39E-20 | 60802 | 0.0085 |
| 4 | 88831249 | rs11934731 | a | g | 0.6738 | -0.0674 | 8.39E-29 | 66623 | 0.0061 |
| 5 | 88288341 | rs7728694 | t | g | 0.461 | -0.0503 | 1.30E-17 | 66527 | 0.0059 |
| 5 | 112221869 | rs818427 | t | c | 0.3118 | 0.0342 | 2.37E-08 | 66592.9 | 0.0061 |
| 5 | 122847622 | rs11745493 | a | g | 0.7463 | 0.0445 | 7.75E-12 | 66597 | 0.0065 |
| 6 | 44636919 | rs7741085 | t | c | 0.5874 | 0.0423 | 1.51E-13 | 66441.1 | 0.0057 |
| 6 | 127167072 | rs13204965 | a | c | 0.771 | 0.0619 | 1.02E-18 | 66132.9 | 0.007 |
| 6 | 151910126 | rs6557155 | t | g | 0.4318 | -0.0751 | 2.56E-37 | 66602 | 0.0059 |
| 6 | 151971720 | rs7740042 | a | t | 0.2024 | -0.0494 | 2.71E-12 | 66602 | 0.0071 |
| 7 | 27989403 | rs757138 | t | g | 0.6889 | -0.0348 | 3.33E-08 | 66043.1 | 0.0063 |
| 7 | 30997087 | rs73305797 | a | t | 0.7569 | 0.0422 | 2.40E-10 | 66180 | 0.0067 |
| 7 | 38142840 | rs34102936 | a | g | 0.5897 | 0.0471 | 1.87E-16 | 66579 | 0.0057 |
| 7 | 50901491 | rs1548607 | a | g | 0.687 | 0.0363 | 4.18E-08 | 66564 | 0.0066 |
| 7 | 96134115 | rs6465511 | c | g | 0.3248 | -0.0738 | 1.03E-34 | 66612 | 0.006 |
| 7 | 96660132 | rs6960249 | t | g | 0.5909 | 0.0325 | 1.45E-08 | 66292 | 0.0057 |
| 7 | 99130834 | rs34670419 | t | g | 0.0394 | -0.088 | 1.09E-08 | 66336 | 0.0154 |
| 7 | 120730944 | rs12534510 | a | c | 0.4455 | -0.0395 | 3.15E-12 | 66614 | 0.0057 |
| 7 | 120974765 | rs3801387 | a | g | 0.7279 | -0.1347 | 1.15E-100 | 66004 | 0.0063 |
| 7 | 121191251 | rs73719807 | a | c | 0.9129 | -0.0925 | 1.14E-16 | 66582 | 0.0112 |
| 7 | 150953205 | rs73169678 | a | c | 0.1117 | 0.0619 | 1.05E-11 | 66472 | 0.0091 |
| 8 | 120012700 | rs11995824 | c | g | 0.4319 | 0.0675 | 1.06E-31 | 66121 | 0.0058 |
| 9 | 54412493 | rs1159798 | a | c | 0.2402 | 0.0429 | 1.01E-09 | 60898 | 0.007 |
| 9 | 133471891 | rs10901216 | a | g | 0.3438 | -0.0474 | 5.53E-15 | 66354 | 0.0061 |
| 10 | 54423853 | rs12258451 | c | g | 0.8693 | 0.0702 | 2.41E-15 | 66519 | 0.0089 |
| 10 | 112245400 | rs73349318 | a | t | 0.8738 | -0.0472 | 2.68E-08 | 66341 | 0.0085 |
| 10 | 124015986 | rs10788264 | a | g | 0.4815 | -0.0338 | 2.61E-09 | 66565 | 0.0057 |
| 11 | 242859 | rs55781332 | a | g | 0.7831 | -0.0552 | 8.07E-16 | 66198 | 0.0069 |
| 11 | 15708792 | rs7926837 | a | g | 0.7854 | -0.0564 | 3.98E-16 | 66568 | 0.0069 |
| 11 | 15816918 | rs10832520 | a | t | 0.0394 | 0.1123 | 1.00E-12 | 66628 | 0.0158 |
| 11 | 16630779 | rs35199438 | t | g | 0.3035 | -0.0489 | 2.36E-15 | 66609.9 | 0.0062 |
| 11 | 27306364 | rs7105860 | c | g | 0.6025 | -0.0468 | 2.36E-15 | 66376 | 0.0059 |
| 11 | 35083633 | rs2553773 | c | g | 0.4137 | -0.037 | 1.49E-10 | 66619 | 0.0058 |
| 11 | 46766890 | rs61884327 | t | c | 0.9022 | -0.0801 | 4.64E-16 | 66175 | 0.0099 |
| 11 | 47284279 | rs143187557 | t | c | 0.0223 | -0.1237 | 1.15E-09 | 66321 | 0.0203 |
| 11 | 68218290 | rs11228240 | t | c | 0.2574 | -0.083 | 1.72E-35 | 66583 | 0.0067 |
| 11 | 86887931 | rs634277 | a | g | 0.6678 | 0.0607 | 2.15E-23 | 66585 | 0.0061 |
| 11 | 121913230 | rs725670 | a | g | 0.383 | -0.0322 | 3.61E-08 | 66565.9 | 0.0059 |
| 12 | 1639249 | rs35125553 | a | g | 0.7145 | -0.0383 | 5.20E-09 | 66278 | 0.0066 |
| 12 | 49379537 | rs118115924 | t | g | 0.0139 | -0.2822 | 6.99E-21 | 58918 | 0.0301 |
| 12 | 49655948 | rs117557198 | a | g | 0.9324 | -0.0769 | 1.58E-10 | 66523 | 0.012 |
| 12 | 53743064 | rs10735851 | a | g | 0.7083 | -0.0541 | 5.84E-18 | 66566 | 0.0063 |
| 12 | 90334829 | rs10777212 | t | g | 0.3455 | 0.0452 | 5.05E-14 | 66619 | 0.006 |
| 12 | 107302778 | rs1037011 | t | c | 0.4792 | -0.0404 | 1.54E-12 | 66616 | 0.0057 |
| 13 | 42952145 | rs9594738 | t | c | 0.4592 | -0.0614 | 3.84E-27 | 66157 | 0.0057 |
| 13 | 43200103 | rs78667121 | a | g | 0.0325 | 0.1326 | 1.70E-13 | 66346 | 0.018 |
| 14 | 91464890 | rs1286150 | t | c | 0.8045 | -0.0549 | 2.44E-14 | 66573 | 0.0072 |
| 15 | 38340874 | rs12442242 | a | g | 0.8501 | -0.0509 | 4.94E-10 | 66418 | 0.0082 |
| 15 | 51537806 | rs2414098 | t | c | 0.3898 | -0.0329 | 1.99E-08 | 66562.1 | 0.0059 |
| 15 | 67547301 | rs3743347 | a | c | 0.2351 | 0.0519 | 1.75E-14 | 66615.1 | 0.0068 |
| 16 | 392318 | rs8047501 | a | g | 0.4923 | 0.0524 | 1.13E-18 | 66340 | 0.0059 |
| 16 | 86714715 | rs71390846 | c | g | 0.1836 | -0.0484 | 1.38E-10 | 65285 | 0.0075 |
| 17 | 2064702 | rs2873195 | a | t | 0.3127 | -0.0406 | 4.31E-11 | 66572 | 0.0062 |
| 17 | 17804725 | rs8070128 | t | c | 0.5763 | -0.0394 | 1.98E-11 | 66625 | 0.0059 |
| 17 | 41819562 | rs144691710 | a | g | 0.9249 | -0.1017 | 2.24E-19 | 66392 | 0.0113 |
| 17 | 42283037 | rs9910055 | t | c | 0.2624 | 0.0442 | 3.12E-11 | 66576.1 | 0.0067 |
| 17 | 60054857 | rs884205 | a | c | 0.2421 | -0.0531 | 4.39E-15 | 66040 | 0.0068 |
| 17 | 63771079 | rs9972944 | a | g | 0.4049 | 0.0363 | 6.87E-10 | 66595 | 0.0059 |
| 20 | 10640877 | rs6040063 | a | g | 0.5007 | 0.0359 | 1.78E-10 | 66499 | 0.0056 |
| 20 | 39103882 | rs6029130 | t | c | 0.2874 | 0.0348 | 3.50E-08 | 66497 | 0.0063 |
| 21 | 28773868 | rs1452102 | t | g | 0.5871 | -0.0345 | 1.74E-09 | 66489 | 0.0057 |
| 21 | 36970350 | rs9976876 | t | g | 0.447 | -0.0375 | 8.01E-11 | 66514 | 0.0058 |
| 21 | 40350744 | rs11910328 | a | g | 0.8351 | -0.0429 | 2.99E-08 | 66298 | 0.0077 |

**Supplemental Table 3 Footnote:** Chr: Chromosome; Pos: position; rsid: variant ID; EA: effect allele; OA: other allele; EAF: effect allele frequency; pval: P-value; StdErr: standard error; N: sample size.

**Supplemental Table 4: Mendelian randomization analysis results in the main and the sensitivity analyses (exposure=bone mineral density; outcome=cardiac metric)**

| **Metric** | **IVW Beta** | **IVW**  **SE** | **IVW pval** | **Weighted  median** | **Weighted  median_pval** | **Weighted mode** | **Weighted  mode_pval** | **MR-Egger** | **MR-Egger  pval** | **egger  intercept** | **egger intercept pval** |
| --- | --- | --- | --- | --- | --- | --- | --- | --- | --- | --- | --- |
| Max atrial area diameter I (7) | 0.06 | 0.018 | 0.001 | 0.05 | 0.031 | 0.04 | 0.342 | 0.03 | 0.4 | 0.001 | 0.6 |
| LVSV(7) | -0.061 | 0.020 | 0.002 | -0.04 | 0.06 | -0.03 | 0.307 | -0.02 | 0.6 | -0.002 | 0.4 |
| RVEDV(7) | -0.056 | 0.018 | 0.003 | -0.04 | 0.032 | -0.03 | 0.302 | -0.08 | 0.07 | 0.001 | 0.4 |
| Pulmonary artery aorta (7) | -0.058 | 0.019 | 0.004 | -0.05 | 0.077 | -0.03 | 0.408 | -0.06 | 0.2 | 0.0001 | 0.9 |
| RVESV(7) | -0.052 | 0.018 | 0.005 | -0.05 | 0.005 | -0.07 | 0.056 | -0.12 | 0.01 | 0.004 | 0.1 |
| Max LV(7) | -0.052 | 0.019 | 0.009 | -0.04 | 0.058 | -0.03 | 0.416 | -0.03 | 0.5 | -0.001 | 0.6 |
| RVSV(7) | -0.049 | 0.018 | 0.009 | -0.01 | 0.404 | 0.001 | 0.957 | -0.02 | 0.5 | -0.001 | 0.6 |
| RVEDV(2) | -0.066 | 0.025 | 0.01 | -0.09 | 0.003 | -0.102 | 0.053 | -0.153 | 0.023 | 0.005 | 0.15 |
| RVESV I (7) | -0.047 | 0.018 | 0.012 | -0.05 | 0.015 | -0.08 | 0.069 | -0.12 | 0.01 | 0.004 | 0.07 |
| RVEDV I (7) | -0.046 | 0.018 | 0.014 | -0.04 | 0.05 | -0.06 | 0.119 | -0.06 | 0.1 | 0.001 | 0.6 |
| RVESV (2) | -0.055 | 0.023 | 0.019 | -0.04 | 0.11 | -0.02 | 0.7 | -0.16 | 0.008 | 0.006 | 0.05 |
| RVEF LVEF ratio (7) | 0.045 | 0.020 | 0.031 | 0.05 | 0.071 | 0.07 | 0.097 | 0.1 | 0.05 | -0.003 | 0.2 |
| RVSV (2) | -0.054 | 0.025 | 0.031 | -0.04 | 0.198 | -0.04 | 0.42 | -0.08 | 0.22 | 0.001 | 0.66 |
| Fractional area change (7) | 0.043 | 0.020 | 0.039 | 0.08 | 0.003 | 0.08 | 0.051 | 0.12 | 0.02 | -0.004 | 0.1 |
| Pulmonary artery aorta diastole (7) | -0.037 | 0.019 | 0.055 | -0.03 | 0.242 | -0.01 | 0.802 | -0.02 | 0.5 | -0.0005 | 0.8 |
| Right atrial min area (7) | -0.036 | 0.019 | 0.057 | -0.03 | 0.126 | -0.04 | 0.288 | -0.12 | 0.01 | 0.005 | 0.06 |
| LVSV I (7) | -0.04 | 0.021 | 0.066 | -0.03 | 0.17 | -0.04 | 0.33 | 0.01 | 0.7 | -0.003 | 0.2 |
| Short axis cm pulmonary artery (7) | -0.037 | 0.021 | 0.08 | -0.03 | 0.273 | -0.09 | 0.068 | -0.1 | 0.07 | 0.003 | 0.2 |
| RVESV LVESV ratio (7) | -0.038 | 0.021 | 0.081 | -0.06 | 0.032 | -0.05 | 0.196 | -0.12 | 0.02 | 0.005 | 0.09 |
| Min LV(7) | -0.031 | 0.018 | 0.093 | -0.01 | 0.543 | 0.008 | 0.876 | -0.01 | 0.7 | -0.0008 | 0.7 |
| RVSV I (7) | -0.032 | 0.019 | 0.103 | -0.02 | 0.347 | -0.01 | 0.689 | 0.007 | 0.8 | -0.0042 | 0.4 |
| Max LV I (7) | -0.034 | 0.021 | 0.117 | -0.04 | 0.13 | -0.08 | 0.062 | 0.007 | 0.9 | -0.002 | 0.4 |
| Arterial stiffness index (4) | 0.016 | 0.010 | 0.118 | 0.01 | 0.28 | 0.01 | 0.36 | 0.03 | 0.1 | -0.001 | 0.3 |
| Ascending  aorta maximum area (5) | 0.03 | 0.019 | 0.119 | 0.01 | 0.682 | -0.03 | 0.441 | -0.01 | 0.7 | 0.002 | 0.3 |
| LVEF (3) | -0.035 | 0.027 | 0.198 | -0.01 | 0.69 | 0.018 | 0.8 | 0.06 | 0.43 | -0.005 | 0.2 |
| Short axis cm root I (7) | 0.023 | 0.017 | 0.2 | -0.01 | 0.653 | -0.02 | 0.562 | -0.02 | 0.5 | 0.003 | 0.2 |
| RVSV LVSV ratio (7) | 0.022 | 0.017 | 0.208 | 0.02 | 0.336 | 0.03 | 0.394 | 0.008 | 0.8 | 0.0008 | 0.7 |
| Ascending  aorta distensibility (5) | 0.024 | 0.018 | 0.208 | 0.03 | 0.251 | 0.03 | 0.472 | -0.01 | 0.8 | 0.002 | 0.3 |
| LVMVR (3) | 0.034 | 0.027 | 0.209 | 0.03 | 0.41 | 0.04 | 0.5 | -0.05 | 0.4 | 0.005 | 0.2 |
| RVEF (7) | 0.023 | 0.018 | 0.223 | 0.04 | 0.09 | 0.08 | 0.035 | 0.12 | 0.009 | -0.006 | 0.01 |
| Right atrial max area (7) | -0.023 | 0.019 | 0.226 | -0.04 | 0.076 | -0.05 | 0.231 | -0.08 | 0.1 | 0.003 | 0.2 |
| LVEDV (3) | -0.04 | 0.033 | 0.231 | -0.06 | 0.11 | -0.1 | 0.12 | 0.01 | 0.8 | -0.002 | 0.5 |
| Ascending aorta minimum area (5) | 0.023 | 0.019 | 0.237 | 0.02 | 0.366 | -0.02 | 0.685 | -0.005 | 0.9 | 0.006 | 0.5 |
| Short axis cm root (7) | -0.019 | 0.018 | 0.295 | -0.01 | 0.514 | -0.01 | 0.775 | -0.06 | 0.16 | 0.002 | 0.2 |
| Short axis cm pulmonary artery diastole I (7) | 0.018 | 0.020 | 0.374 | 0.003 | 0.892 | -0.04 | 0.468 | -0.01 | 0.7 | 0.002 | 0.5 |
| Min LV I (7) | -0.017 | 0.019 | 0.387 | -0.02 | 0.29 | -0.05 | 0.351 | 0.006 | 0.9 | -0.001 | 0.6 |
| Max atrial area diameter (7) | 0.016 | 0.018 | 0.4 | -0.01 | 0.58 | -0.02 | 0.513 | -0.04 | 0.4 | 0.003 | 0.2 |
| Minimum ascending aortic area(1) | 2.571 | 3.13 | 0.412 | -1.68 | 0.7 | -8.76 | 0.279 | -7.85 | 0.3 | 0.6 | 0.1 |
| Right atrial min area I (7) | -0.015 | 0.018 | 0.422 | -0.01 | 0.635 | -0.02 | 0.659 | -0.09 | 0.04 | 0.004 | 0.06 |
| Minimum descending aortic area (1) | 1.203 | 1.50 | 0.424 | 2.05 | 0.276 | 1.49 | 0.754 | -4.8 | 0.2 | 0.3 | 0.09 |
| LVEF (7) | -0.014 | 0.018 | 0.434 | -0.01 | 0.775 | -0.004 | 0.914 | 0.002 | 0.9 | -0.0009 | 0.7 |
| Short axis cm pulmonary artery diastole (7) | -0.016 | 0.020 | 0.448 | -0.03 | 0.145 | -0.09 | 0.025 | -0.05 | 0.2 | 0.002 | 0.4 |
| Maximum  ascending aortic area (1) | 2.415 | 3.23 | 0.455 | -1.4 | 0.756 | -7.02 | 0.382 | -7.9 | 0.3 | 0.6 | 0.1 |
| RVEF (2) | 0.016 | 0.021 | 0.455 | 0.004 | 0.89 | 0.07 | 0.22 | 0.09 | 0.09 | -0.004 | 0.13 |
| LA active  emptying fraction (6) | 0.014 | 0.019 | 0.461 | 0.03 | 0.229 | 0.05 | 0.267 | 0.01 | 0.7 | 7.57E-05 | 0.9 |
| Maximum  descending aortic area (1) | 1.132 | 1.60 | 0.48 | 0.85 | 0.685 | -5.44 | 0.215 | -5 | 0.2 | 0.3 | 0.1 |
| RVEDV LVEDV ratio (7) | -0.013 | 0.019 | 0.526 | 0.006 | 0.818 | 0.0001 | 0.997 | -0.07 | 0.1 | 0.003 | 0.2 |
| Indexed LA maximum volume (6) | -0.012 | 0.019 | 0.528 | -0.006 | 0.818 | 0.01 | 0.817 | 0.07 | 0.1 | -0.005 | 0.05 |
| Short axis cm pulmonary artery strain (7) | 0.011 | 0.021 | 0.601 | 0.05 | 0.101 | 0.07 | 0.092 | 0.04 | 0.4 | -0.002 | 0.5 |
| Indexed  LA minimum volume (6) | -0.009 | 0.019 | 0.652 | 0.01 | 0.521 | 0.03 | 0.513 | 0.05 | 0.2 | -0.003 | 0.1 |
| LVM (3) | -0.014 | 0.031 | 0.658 | -0.03 | 0.48 | -0.03 | 0.6 | -0.02 | 0.8 | 0.0005 | 0.9 |
| Right atrial max area I (7) | 0.007 | 0.018 | 0.715 | -0.01 | 0.639 | -0.05 | 0.228 | -0.03 | 0.4 | 0.002 | 0.3 |
| LA passive  emptying fraction (6) | 0.006 | 0.019 | 0.751 | -0.006 | 0.828 | -0.008 | 0.839 | -0.008 | 0.8 | 0.0009 | 0.7 |
| LA total  emptying fraction (6) | 0.005 | 0.018 | 0.79 | 0.009 | 0.747 | 0.02 | 0.591 | 0.004 | 0.9 | 4.39E-05 | 0.9 |
| LVESV (3) | -0.007 | 0.031 | 0.814 | -0.01 | 0.71 | -0.09 | 0.2 | -0.05 | 0.5 | 0.002 | 0.5 |
| Short axis cm pulmonary artery I (7) | 0.003 | 0.019 | 0.878 | 0.006 | 0.807 | -0.01 | 0.728 | -0.05 | 0.2 | 0.003 | 0.2 |
| Descending  aortic distensibility (1) | 0.001 | 0.015 | 0.934 | 0.01 | 0.617 | 0.05 | 0.158 | 0.06 | 0.1 | -0.003 | 0.1 |
| Ascending  aortic distensibility (1) | -0.001 | 0.017 | 0.963 | 0.03 | 0.123 | 0.07 | 0.05 | 0.03 | 0.4 | -0.002 | 0.4 |

**Supplemental Table 4 footnote:** The number enclosed in brackets indicates the source study from which these metrics were derived, as detailed in Supplementary Table 2. pval, p value; SE, standard error; IVW, Inverse variance weighted; I, indexed; LV, Left ventricle; LVSV, LV stroke volume; RV, right ventricle; RVEDV, RV end-diastolic volume; RVESV, RV end-systolic volume; RVSV, right ventricle stroke volume; RVEDV, right ventricle end-diastolic volume; EVEF, RV ejection fraction; LVEF, LV ejection fraction; cm, centimetre; Max LV I, maximum LV indexed; LVM, LV mass; LVMVR, LV mass to volume ratio; LA, left atrium; LVESV, LV end-systolic volume.

**Supplemental Table 5: Associations between baseline heel eBMD and prevalent CVDs stratified by sex, based on Model 3 estimates**

| **Outcome** | **Women**  **n=** **216,514** | **Men**  **n=** **182,783** | **p interaction** |
| --- | --- | --- | --- |
|  | *OR (95% CI)* | |  |
| Ischaemic heart disease | 0.97 (0.93,0.99) | 0.98 (0.96,1.00) | 0.43 |
| Myocardial infarction | 0.91 (0.87,0.96) | 0.97 (0.95,0.97) | 0.02 |
| Heart failure | 0.82 (0.74,0.91) | 0.86 (0.81,0.91) | 0.47 |
| Non-ischaemic cardiomyopathies | 0.79 (0.67,0.92) | 0.85 (0.78,0.93) | 0.40 |
| Arrhythmia | 0.88 (0.81,0.96) | 0.98 (0.93,1.04) | 0.02 |

**Supplemental Table 5 footnote:** Results are reported as odds ratios (ORs) per 1-standard deviation (SD) increment of eBMD. Model 3 is adjusted for age, sex, diabetes, hypertension, high cholesterol, smoking status, body mass index, alcohol intake frequency, physical activity, Townsend score, and educational level and BMI. The total number of patients in Model 3 is 399,297. eBMD, estimated bone mineral density; CVD, cardiovascular disease; CI, confidence interval.

**Supplemental Table 6: Associations between baseline heel eBMD and incident CVDs stratified by sex, based on Model 3 estimates**

| **Outcome** | **Women**  **n=** **216,514** | **Men**  **n=** **182,783** | **p interaction** |
| --- | --- | --- | --- |
|  | **SRH (95%CI)** | |  |
| Ischaemic heart disease | 0.95 (0.93,0.98) | 1.02 (1.00,1.04) | <0.001 |
| Myocardial infarction | 0.98 (0.94,1.02) | 1.02 (0.99,1.05) | 0.001 |
| Cardiomyopathies | 0.88 (0.82,0.94) | 0.99 (0.94,1.05) | 0.002 |
| Heart failure | 0.84 (0.81,0.87) | 0.94 (0.91,0.96) | <0.001 |
| Arrhythmia | 0.91 (0.88,0.93) | 0.98 (0.97,1.00) | <0.001 |

**Supplemental Table 6 footnote:** Results are reported as sub-distribution hazard ratios (SHR) per 1-standard deviation (SD) increment of BMD. Model 3 is adjusted for age, sex, diabetes, hypertension, high cholesterol, smoking status, body mass index, alcohol intake frequency, physical activity, Townsend score, and educational level and BMI. The total number of patients in Model 3 is 399,297. eBMD, estimated bone mineral density; CVD, cardiovascular disease; CI, confidence interval.

**Supplemental Table 7: Associations between baseline heel eBMD and mortality stratified by sex, based on Model 3 estimates**

| **Outcome** | **Women**  **n=** **216,514** | **Men**  **n=** **182,783** | **p interaction** |
| --- | --- | --- | --- |
|  | **HR (95%CI)** | |  |
| All-cause mortality | 0.89 (0.87,0.91) | 0.85 (0.84,0.87) | <0.001 |
| CVD mortality | 0.85 (0.82,0.89) | 0.85 (0.83,0.88) | 0.56 |
| IHD mortality | 0.87 (0.81,0.95) | 0.88 (0.84,0.92) | 0.35 |

**Supplemental Table 7 footnote:** Results are reported as hazard ratios (HR) per 1-standard deviation (SD) increment of eBMD. Model 3 is adjusted for age, sex, diabetes, hypertension, high cholesterol, smoking status, body mass index, alcohol intake frequency, physical activity, Townsend score, and educational level and BMI. The total number of patients in Model 3 is 399,297. eBMD, estimated bone mineral density; CVD, cardiovascular disease; CI, confidence interval.

**Supplemental references**

1. Francis CM, Futschik ME, Huang J, et al. Genome-wide associations of aortic distensibility suggest causality for aortic aneurysms and brain white matter hyperintensities. *Nat Commun.* 2022;13(1):4505. doi: 10.1038/s41467-022-32219-x
2. Aung N, Vargas JD, Yang C, et al. Genome-wide association analysis reveals insights into the genetic architecture of right ventricular structure and function. *Nat Genet.* 2022;54(6):783-791. doi: 10.1038/s41588-022-01083-2
3. Aung N, Vargas JD, Yang C, et al. Genome-Wide Analysis of Left Ventricular Image-Derived Phenotypes Identifies Fourteen Loci Associated With Cardiac Morphogenesis and Heart Failure Development. *Circulation.* 2019;140(16):1318-1330. doi: 10.1161/CIRCULATIONAHA.119.041161
4. Fung K, Ramírez J, Warren HR, et al. Genome-wide association study identifies loci for arterial stiffness index in 127,121 UK Biobank participants. *Sci Rep.* 2019;9(1):9143. doi: 10.1038/s41598-019-45703-0
5. Benjamins JW, Yeung MW, van de Vegte YJ, et al. Genomic insights in ascending aortic size and distensibility. *EBioMedicine*. 2022;75:103783. doi: 10.1016/j.ebiom.2021.103783.
6. Ahlberg G, Andreasen L, Ghouse J, et al. Genome-wide association study identifies 18 novel loci associated with left atrial volume and function. *Eur Heart J*. 2021;42(44):4523-4534. doi: 10.1093/eurheartj/ehab466
7. Pirruccello JP, Di Achille P, Nauffal V, et al. Genetic analysis of right heart structure and function in 40,000 people. *Nat Genet*. 2022;54(6):792-803. doi: 10.1038/s41588-022-01090-3
